# Retrospective study of 289 odontogenic tumors in a Brazilian population

**DOI:** 10.4317/medoral.21029

**Published:** 2016-01-31

**Authors:** Leorik-Pereira da Silva, Marianna-Sampaio Serpa, Jefferson-da-Rocha Tenório, George-João-Ferreira do Nascimento, Emanuel-Sávio de Souza-Andrade, Ana-Paula Veras-Sobral

**Affiliations:** 1 DDS. Msc student, Oral Pathology, Departament of Dentistry, Federal University of Rio Grande do Norte - UFRN, Natal, Rio Grande do Norte, Brazil; 2 DDS. MSc. PhD. Adjunct Professor, Oral Pathology, Biological Sciences Academic Center, School of Dentistry, Federal University of Campina Grande - UFCG, Patos, Paraíba, Brazil; 3 DDS. MSc. PhD. Associated Professor, Oral Pathology, School of Dentistry, University of Pernambuco, Camaragibe, Pernambuco, Brazil

## Abstract

**Background:**

Odontogenic tumors (OTs) are considered important among oral lesions because of their clinicopathological heterogeneity, and variable biological behavior. This paper aims to determine the frequency and distribution of OTs, over a period of 10 years, at a public university in Northeastern Brazil and compare this data with previous reports.

**Material and Methods:**

We reviewed all cases of OTs from oral pathology laboratory of University of Pernambuco (UPE), from 2004 to 2014. Diagnoses were re-evaluated and the tumors were classified according to the latest (2005) World Health Organization Classification of Tumors. In addition, we searched in the English-language literature retrospective studies on OTs that used the same classification.

**Results:**

Data was obtained allowing the analysis of the tissue hemodynamics. We were able to map the vascularization of the face and it was possible to access three arteries of small diameter (0,60mm angular artery; 0,55mm greater palatine artery; 0,45mm infraorbital artery).

**Conclusions:**

OTs are uncommon neoplasms with geographic variation. Our clinicopathological features are according to literature. In the present study, KCOT was the most frequent one, showing that the new classification of OTs altered the distribution of these lesions and possibly made KCOT the most common OT observed in diagnostic services worldwide.

**Key words:**Odontogenic tumors, jaw neoplasms, epidemiology, oral pathology.

## Introduction

Odontogenic tumors (OTs) constitute a complex group of tumors that have heterogeneous clinical behavior and several histological types. Some of these lesions are true neoplasms and may rarely present a malignant behavior; and some are similar to tumors malformations, namely hamartomas. Due to the diversity of lesions that can arise from odontogenic tissues, several classification systems have been published in an attempt to define diagnostic criteria (1-5).

The first internationally accepted classification of OTs was published in 1971 by the World Health Organization (WHO) and then updated in 1992. Due to advances in immunohistochemistry and molecular biology during the last decade a revision of the 1992 edition was proposed by Philipsen and Reichart and published in 2005. The WHO classified the OTs in 3 groups: Odontogenic epithelium with mature fibrous stroma, without odontogenic ectomesenchyme; Odontogenic epithelium with odontogenic ectomesenchyme, with or without hard tissue formation; and Mesenchyme and/or odontogenic ectomesenchyme, with or without odontogenic epithelium. In this new classification, keratocystic odontogenic tumor (KCOT), previously classified as a cystic lesion, was included as a tumor. This re-categorization was made to highlight its aggressive behavior and high recurrence (1-3,5).

The frequency and incidence of some OTs are controversial and depends in the geographic location. The aim of this study was to determine the epidemiology of this heterogeneous group of lesions over a period of 10 years at a public university in Northeastern Brazil and to compare this data with those in literature.

## Material and Methods

The files and histological material was obtained from the oral pathology laboratory, School of Dentistry, University of Pernambuco, Northeastern Brazil. Case reports of patients with OTs over a 10-year period (2004-2014) were used. This retrospective study was approved by the Research Ethics Committee of the University of Pernambuco (protocol nº 163.794).

To evaluate the incidence of OTs in the region the sample size was the population of Pernambuco-Brazil according to the annual averaged populations for 2014, reported by the Brazilian Institute of Geography and Statistics as 9.27 million.

Cases diagnosed histopathologically as OT were retrieved from the laboratory for review and were reevaluated according to the 2005 WHO Classification of OTs (5). Slides without histopathologic criteria for definitive diagnosis of OT and cases without slides and paraffin-embedded tumor specimens were excluded. Data were analyzed for age, gender, tumor site and histologic type. In addition, we searched on PubMed Database English-language series of cases using the 2005 WHO classification to compare their data with ours.

After the sample was obtained, a database was generated using commercially available software (SPSS 13.0). Continuous variables were categorized to facilitate data analysis and presentation. Gender and tumor site analyses were done using the binomial test. X2 test was applied to check the statistical signifi-cance of the findings. The level of significance adopted was *p*< 0.05.

## Results

Among the 6,028 oral biopsies registered during the 10-year period of this retrospective study, 302 cases were diagnosed as OT. After reevaluating the hematoxylin and eosin-stained sections and data, 13 cases were excluded, leaving a total of 289 cases which constitutes 4.8% of all biopsies conducted during this period. Of the excluded cases, 5 were rejected because they did not satisfy the 2005 WHO criteria and 6 lacked slides/paraffin-embedded tumor specimens or data. Two cases of orthokeratinized odontogenic cyst were excluded on the basis of the classification used.

The overall incidence of OTs in the Pernambuco population in the current study was 31.1/million. Tumors of odontogenic epithelium had an incidence of 22.5/million people, followed by mixed tumors (6.5/million) and mesenchyme tumors (1.8/million). Malignant tumors had an incidence of 0.02/million.

Of the 289 cases of OTs, 285 (98.6%) were intraosseous and four (1.4%) were extraosseous (3 peripheral ameloblastomas and 1 peripheral calcifying epithelial odontogenic tumor). There were 287 benign (99.3%) and only two malignant tumors were found (0.7%), one ameloblastic carcinoma and one ameloblastic fibrodentinosarcoma. Tumors of odontogenic epithelium with mature fibrous stroma without odontogenic ectomesenchyme were the most common with 209 cases (72.3%).

The most frequent tumor was KCOT (34.6%), followed by ameloblastoma (AMB) (32.9%) and odontoma (ODO) (11.4%). Statistical analysis revealed no significant difference between the proportions of these three groups (*p*= 0 .783). The age of patients ranged from 3 to 84 years, with a mean of 35 years (s.d. = 15.3 years), most often affecting patients between the second and fourth decades ( Table 1).

**Table 1:**
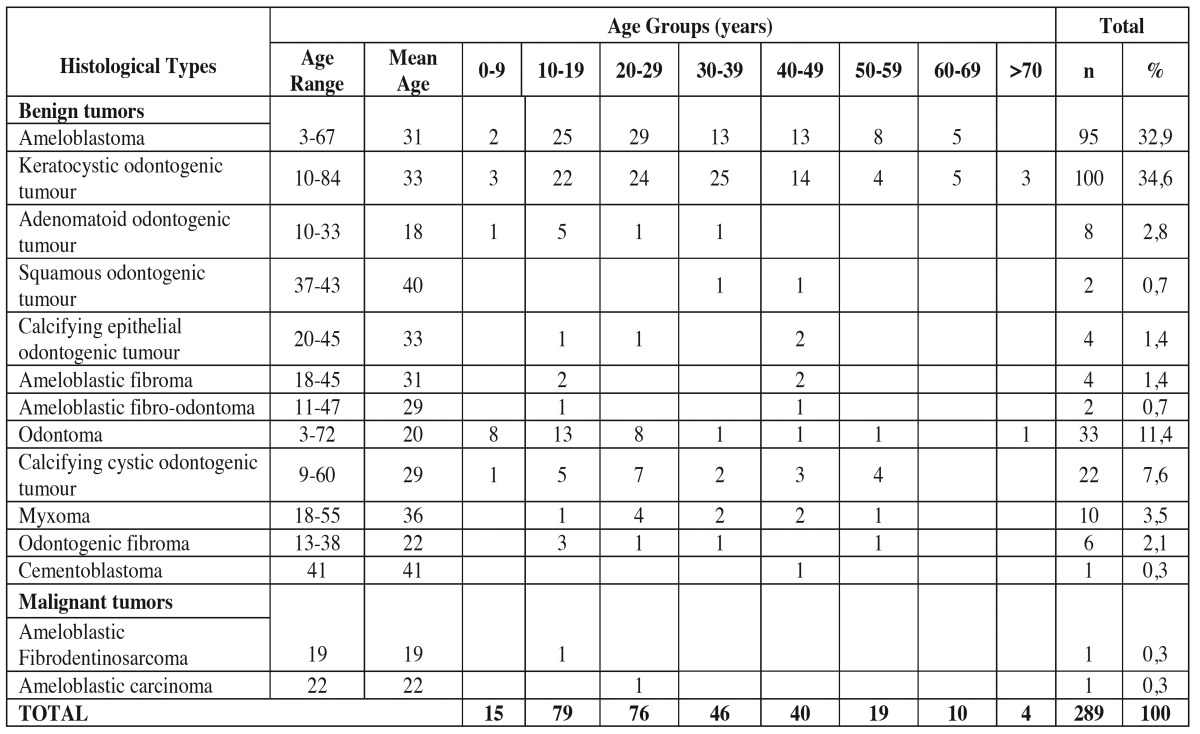
Age distribution of odontogenic tumors by decades.

Regarding gender, 56.4% of all tumors occurred in females and 43.6% in males. Statistical analysis revealed no significant difference in the distribution of OT in relation to gender (*p*=0.743). The female-male ratio was 1.3:1. The mandible (206 cases, 71.3%) was 2.5 times more commonly affected than the maxilla (83 cases; 28.7%), and this was particularly remarkable for AMB and KCOT (*p*= 0.024) ( Table 2).

**Table 2:**
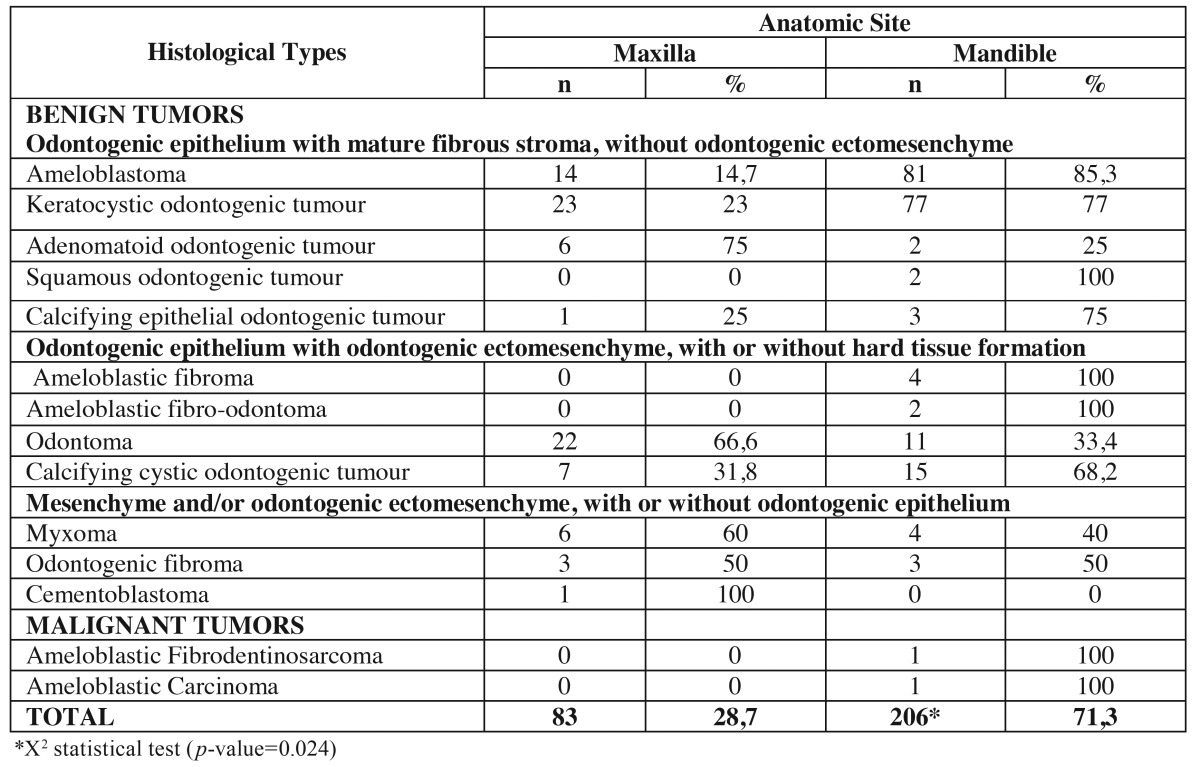
Site distribution of benign and malignant odontogenic tumors.

## Discussion

OTs are jaws lesions relatively uncommon with great clinical and histopathological diversity which reflects its distinguish biological behavior. The epidemiological profile and histological classification must be considered when these lesions are studied. Few of the published works studied a big amount of cases evaluating the incidence of OTs in a country or area using WHO current classification (5).

The incidence of OTs in the present study was 4.8% of all specimens recorded between 2004 and 2014. This data is higher than previously studies reported in North America (6,7), South America (8-12) and Europe (13,14), as they presented a frequency lower than 3%. On the other hand, in Asia and Africa OTs represented 8.99 and 9.6%, respectively, of all oral lesions (15,16), although an Iranian series had a frequency of 1.9% (17).

Comparing the incidence of our study with other reports in Brazil, we observed that OTs present a distinct frequency in different regions. Furthermore, incidence herein obtained was higher (4.8%) than that found in other regions, which ranged from 1.3% to 3% (12,18-20). These findings demonstrate that OTs have variable incidence worldwide and may present regional differences in a country with a continental dimension as Brazil.

Corroborating with other studies (12,18-30), in our sample, benign tumors were prevalent. This low frequency of malignant tumors is similar to those reported in others countries (7-12,19), but it is different than those reported in China that present more than 5% of cases as malignant tumors (16-29).

We have observed a greater incidence of OTs in the female gender, result also found by Gaitán-Cepeda *et al.*(30). However, other studies have found a greater incidence in male gender (18,21,22). Regarding age, there was a higher incidence in the second and third decades of life, being rare after the seventh decade. These findings are similar to the ones reported by Luo and Li (16), da-Costa *et al.* (18) and Johnson *et al.* (22). The mandible was 2.5 times more common than the maxilla which is similar to previous studies (8,18,21,22).

KCOT was the most prevalent OT in this study, followed by AMB and ODO. This finding corroborates with various studies made in different countries, as presented in  Table 3. Data from the literature show differences in the frequencies of these tumors in different countries. Several retrospective studies of OTs published previously to the latest WHO classification (5) reported AMB and ODO being the most common tumors (7,13,19,29). However, the inclusion of KCOT has not only produced an increase in the frequency and prevalence of OTs, but also has modified the order of distribution of these tumors, making KCOT one of the most common OTs (18-22,30).

**Table 3:**
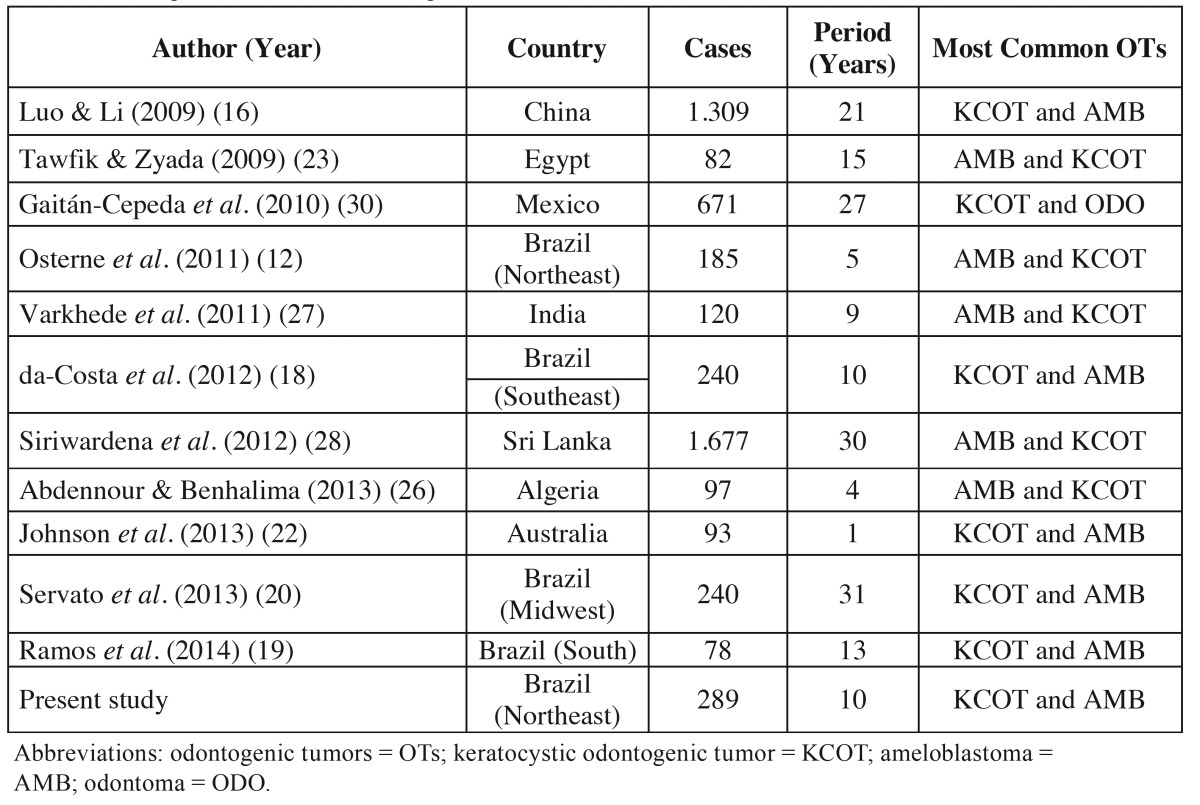
Retrospective studies of odontogenic tumors that considered 2005 WHO classification.

OTs are uncommon lesions in the studied population and are represented mainly by the KCOT, AMB and ODO. Our clinicopathological features are according to literature. Thus, this study shows that the new classification of OTs altered the frequency of the lesions possibly making KCOT the most common OT observed in diagnostic services worldwide.
